# New Possibilities of Field Data Survey in Forest Road Design

**DOI:** 10.3390/s25134192

**Published:** 2025-07-05

**Authors:** Mihael Lovrinčević, Ivica Papa, David Janeš, Luka Hodak, Tibor Pentek, Andreja Đuka

**Affiliations:** 1 Department of Forest Engineering, Faculty of Forestry and Wood Technology, University of Zagreb, Svetošimunska Cesta 23, 10 000 Zagreb, Croatia; mlovrin@sumfak.unizg.hr (M.L.); lhodak@sumfak.unizg.hr (L.H.); tpentek@sumfak.unizg.hr (T.P.); aduka@sumfak.unizg.hr (A.Đ.); 2 Croatian Forests Ltd., Forest Administration Office Delnice, Supilova 32, 51300 Delnice, Croatia; david.janes@hrsume.hr

**Keywords:** theodolite, total station, GNSS, UAV, photogrammetry, lidar, DTM, forest accuracy

## Abstract

Field data, as the basis for planning and designing forest roads, must have high spatial accuracy. Classical (using a theodolite and a level) and modern (based on total stations and GNSSs) surveying methods are used in current field data survey for forest road design. This study analyzed the spatial accuracy of classical and modern surveying methods, the accuracy of spatial data recorded using a UAV equipped with an RGB camera at different flight altitudes, and the accuracy of lidar data of the Republic of Croatia. This study was conducted on a forest area where salvage logging was carried out, which enabled the use of a GNSS receiver in RTK mode as a reference method. The highest RMSE values of the spatial coordinates were recorded for measurements obtained with the classical surveying method (0.89 m) and a total station (0.33 m). The flight altitude of the UAV did not significantly affect the spatial error of the collected data, which ranged between 0.07 and 0.09 m. The cross-terrain slope, as one of the factors that significantly affect the amount of earthworks, did not differ statistically significantly between the methods. The ALS error was strongly influenced by the cross-terrain slope. The authors conclude that the new survey methods (SfM and lidar data) provide high-accuracy data but also draw attention to challenges in their use, such as vegetation and biomass on the ground.

## 1. Introduction

The establishment of an optimal forest road network requires four phases: the planning, design, construction, and management of forest roads [1]. The absence or poor performance of any of the mentioned phases consequently causes various negative impacts on the forest road, the area in which the forest road is located, and the costs of harvesting [2,3,4,5,6]. The forest road design phase can be divided into two separate but mutually dependent subphases: subphase 1 refers to the collection of general and technical data, and subphase 2 is divided into (2a) field surveys and (2b) project development, i.e., office data processing, printing, and results drawing [7]. The office processing of the collected data is carried out with the help of specialized software packages for the design of forest roads, such as RoadEng (Softree Technical Systems). In addition to specialized programs, to design forest roads, programs developed for the design of public roads, such as the Civil 3D software, can also be used. Regardless of the program used, field data, which are the basis for planning and designing a forest road, must be of high spatial accuracy [8]. For field data collection, to design forest roads, various methods of measurement are used in forestry [9]:

- The classical method (using theodolites, levels, and inclinometers);
- The modern method (based on total stations and GNSSs).

The classic method of measurement involves the use of a theodolite, a level, and an inclinometer. The theodolite is the most precise instrument designed for the measurement of horizontal and vertical angles [10]. The total station was developed by combining the angle-measuring capabilities of a theodolite with an electronic distance measurement (EDM) [11]. A total station can determine the horizontal angle, vertical angle, and slope distance to a particular point. A total station, similar to a theodolite, achieves extremely high horizontal and vertical accuracy, with an error of 0.00028–0.00139 degrees [12]. The accuracy of these almost perfect measuring devices can be influenced by factors such as human error (collimation and pointing errors) [13,14] or the calibration and malfunction of the device (optical plummet errors, atmospheric corrections, and the adjustment of the prism poles error) [14]. As the work with the theodolite and the total station is time-consuming [11,15,16], and the lack of labor in forestry and construction is a growing problem [17,18,19], it should not be surprising that GNSS receivers are being implemented in forestry [20,21]. GNSS surveys using a GNSS receiver in real-time kinematics mode (RTK) can achieve subcentimeter accuracy [16,22,23]. Several factors can affect GNSS accuracy: (1) the canopy structure and its density; (2) the tree type; (3) the terrain area; and (4) the field measurement season [24,25,26]. In addition to these measuring devices, data obtained via a photogrammetric analysis or recorded with lidar sensors have been increasingly used in recent times. These methods, as a new way of measuring, could be classified as experimental methods of measurement. The implementation of UAVs in forestry, equipped with sensors for photogrammetric and lidar imaging, is well accepted, primarily due to their autonomy, reliability, safety, and control [27]. Although in certain professions and forestry works, these experimental methods represent “go-to” methods for measurement [28,29,30,31,32], their accuracy needs to be tested for forest road planning and design.

In previous studies on the spatial accuracy of photogrammetric data recorded with UAVs (UAV SfM), the recorded root-mean-square error (RMSE) ranged between 2.3 and 123 cm [33,34,35,36,37,38]. The accuracy of this system can be influenced by several factors, such as flight parameters [39], georeferencing and correction methods [34,36], and lighting [40]. Lepoglavec et al. [41] found no significant differences in the longitudinal slope of the road between a GNSS and the digital terrain model (DTM) developed based on UAV aerial photographs. Depending on the method used to collect lidar data and the sensors themselves, the RMSE of the spatial data can range from 7 cm, for data collected with UAVs, equipped with a lidar sensor [42], and between 12 and 50 cm for data collected with lidar sensors, installed on aircraft [43,44,45]. Kweon et al. [46] determined that the error in the recorded length of measured forest roads with a mobile laser scanner (MLS), compared with a GNSS, ranged from 0.1 to 0.7 m, while the difference between the GNSS and a total station was 2.1 to 4.7 m.

This research tested the spatial accuracy of data recorded using different measurement methods—the classical method; the modern method; the use of an unmanned aerial vehicle equipped with an RGB camera at different flight altitudes; and lidar data, based on airborne laser scanning (ALS)—to collect data necessary for the design of forest roads. Furthermore, as the cross-terrain slope can significantly affect earthwork volumes [47], which, consequently, affect the cost of forest road construction [48], the differences in the obtained cross-terrain slopes were also examined. Finally, the differences in the forest road planned route central axis, measured using different field survey methods, (theodolites, total stations, GNSSs, and UAVs) were tested.

## 2. Materials and Methods

### 2.1. Research Area

**Figure 1:**
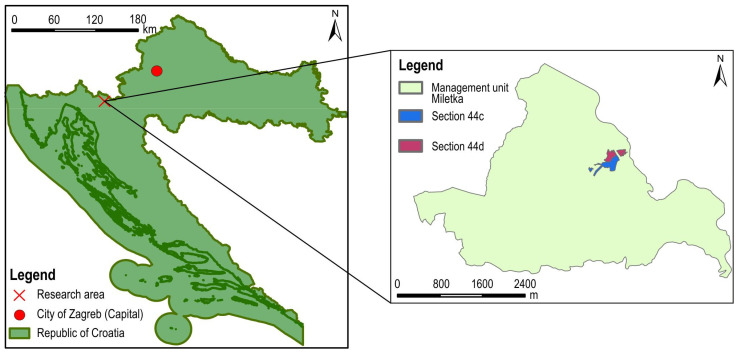
Research area.

**Figure 2:**
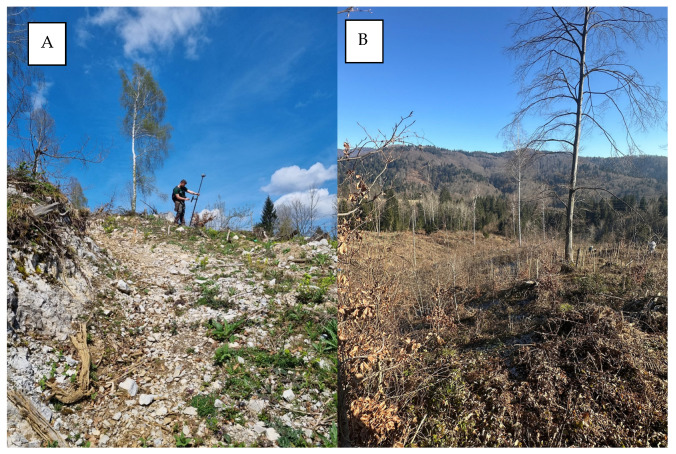
Research area condition. (**A**) Rocky surfaces. (**B**) Biomass in heaps.

This research was conducted in the state forests of the Republic of Croatia managed by the company Croatian Forests Ltd., Forest Administration Delnice, Forest office Vrbovsko, Management Unit Miletka, Sections 44 c and 44 d (Figure 1). The phytocenose of the above forest sections is *Aremonio-Piceetum* (Horvat 1938), where common spruce (*Picea abies* L.) predominates. According to the Mellgren classification [49], the investigated area has a moderately steep terrain (32%). In 2017, salvage logging was carried out in the investigated sections due to attacks from bark beetle (*Ips typographus* L.). After felling, the forest order was established, and for this reason, a large amount of biomass (in heaps) was left on the ground (Figure 2B). As only individual trees were present in the study area, more than 90% of the area was not under tree canopy.

### 2.2. Data Collection

Data collection was carried out using a classical, modern and experimental method of survey. Before the start of the field survey, a zero line was planned on the contour map at M 1:25,000. The maximum longitudinal slope of the used zero line did not exceed 10%, which is fully in accordance with the technical specifications of forest roads in the Republic of Croatia [50,51]. This was followed by the transfer of the planned zero line to the terrain and the fitting of the horizontal alignment polygon, which ultimately represented the final horizontal central axis of the planned forest road. The zero line was placed and the horizontal alignment was decided according to the methods described by Pičman [52]. The set polygon consisted of 30 intersection points (IPs) with a total length of 879.42 m.

#### 2.2.1. Classical Method of Measurement

**Figure 3:**
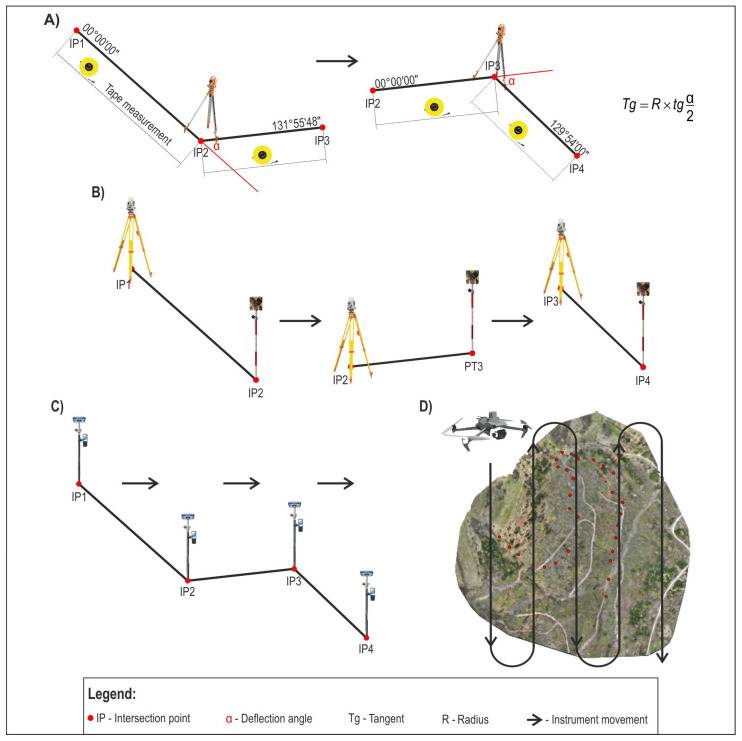
Principles of operation for each measuring device: (**A**) theodolite, (**B**) total station, (**C**) GNSS, and (**D**) UAV SfM.

For this study, a theodolite, a level, and a measuring rod were used as the classical method of survey. The classical survey method was included in this study as it is still used in specific forest conditions such as dense forest areas, where the use of a total station and its EDM can be very limited with a lack of prism (reflector) sight. After placing the horizontal alignment polygon, the deflection angles were measured with a digital theodolite (Geomax ZIPP 02) (Figure 3A), the radii were defined, and the main elements of each horizontal curve were calculated based on these values. This was followed by staking out the horizontal curves, which defined the positions of the beginning, middle, and end of the curve of each horizontal curve, while on the flat parts of the route, where the distance between the end of the curve of the previous horizontal curve and the beginning of the curve of the next horizontal curve was more than 15 m, additional profiles were placed (intermediate points). In this case, the distance of the intermediate point from the previous profile was 10 m. Based on 30 intersection points (IPs), a total of 28 horizontal curves were staked, which also represented the number of times the instrument was moved (Figure 3A). A total of 98 profiles (start, middle, and end points of the horizontal curves and intermediate points) were placed on the planned route of the forest road. The positions of the beginning and end of the arc (horizontal curve) were determined based on the following formula (1) for calculating the tangent value:

(1) $$Tg=R\times tg\frac{\alpha }{2}$$

where

Tg—tangent value (m);

R—selected radius (m);

α—deflection angle (°).

The leveling of the profiles was carried out using a level (Sokkia C330) and a geodetic rod. When leveling the profile, the device was moved 28 times. Terrain cross sections were recorded using two geodetic rods, recording the relative relationships (increase or decrease in the elevation) every two meters perpendicular to the position of the profile with an accuracy of 1 mm. This way, the cross section of the terrain was recorded in a strip of 16 m around the central axis of the planned forest road. The survey with the theodolite and level required two people, while the recording of the terrain cross section required three people.

#### 2.2.2. Modern Method of Measurement

**Figure 4:**
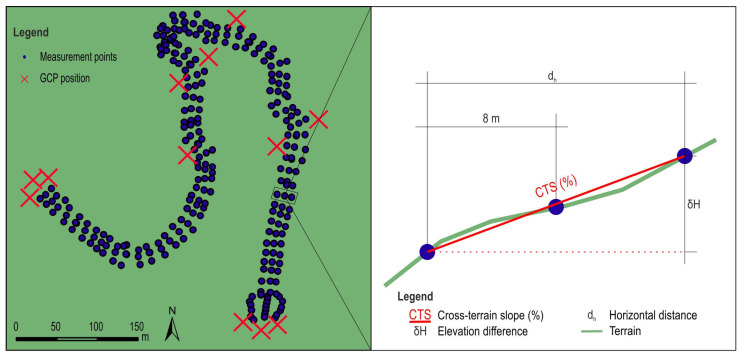
Terrain points of cross-section profiles of planned forest road.

For the modern field survey method, the Stonex R35 total station (Figure 3B) and the Stonex S900A GNSS (Figure 3C) were used. All the profiles placed on the horizontal polygon of the planned forest road and the terrain cross section were recorded at the same points where classic survey method points were recorded. In each profile, three terrain points were recorded with a total station and a GNSS receiver: one on the central axis of the planned forest road route and one each perpendicular to the central axis at a distance of 8 m to the left and right (Figure 4). A total of 250 points were recorded with these devices. In places where certain profiles were almost in the same position (connected horizontal curves in the same or different directions), the points on the central axis were recorded, indicating the position of the profile, and the end of the 8 m band was recorded with one point for both profiles. The total station was moved a total of 28 times. The GNSS was used in RTK mode. The used correction base was the Croatian Positioning System (CROPOS). CROPOS is a system of 59 reference GNSS stations located 70 km from each other. Reference stations collect satellite measurement data and calculate correction parameters available to field users via mobile Internet (GPRS/GSM) [53]. A total station field survey requires two people, while only one is needed for a GNSS receiver survey.

#### 2.2.3. Experimental Method of Measurement

The research area was recorded using a UAV (DJI Mavic 3 Enterprise) (Figure 3D). The flight mission was created in DJI Pilot 2. Four surveys were performed:

- At a height of 60 m with the terrain follow function disabled (60NF);
- At a height of 70 m with the terrain follow function disabled (70NF);
- At a height of 70 m with terrain follow function enabled (70F);
- At a height of 90 m with terrain follow function enabled (90F).

Flying at an altitude of 60 m using the terrain follow option was not possible due to the power line located at the research site. The terrain follow option cannot be turned off when flying at an altitude of 90 m because the flying height in certain parts of the research area would then be higher than 120 m above the ground, which violates the legislation on UAV use in the Republic of Croatia. In all four flight missions, 80% front and 80% side overlaps of aerophotographs were used. During flights, the UAV was equipped with an RTK module, and additional ground control points (GCPs) were placed on the ground (12). GCPs were recorded with a GNSS receiver according to the method followed in Mihelič et al. [54]. Check point markings (a cross on A4 paper), used to analyze the spatial accuracy of the data collected with the UAV, were placed at the same positions where the terrain points were recorded with the classic and modern survey methods.

In addition to UAV surveys, as an experimental survey method, ALS data from the Croatian Geodetic Administration was also tested. ALS data was used in the part of the analyses that required no ground markings. According to the lidar product specification, in areas covered with forests (deciduous and coniferous), the minimum required point density was 4 points/m^2^, with a minimum height accuracy of ±0.1 m (z coordinates) and a horizontal accuracy of ±0.2 m (xy coordinates) [55]. Based on the lidar data, a DTM with a grid width of 1 m (1 × 1 m) (DTM_ALS_) was created. The point elevations (z coordinates) of the created DTM of the planned forest road were extracted in *ArcMap* (v. 10.8) using the Add Surface Information tool. The points recorded by a GNSS receiver were used as positions for z-coordinate extraction.

### 2.3. Data Processing

Classical survey method recordings were entered into the Cesta (v 1.6) software. On the basis of the azimuths, profile heights, and terrain cross-section development, spatial data was obtained. The first and second theodolite survey points were georeferenced based on a GNSS survey.

A photogrammetric analysis of the collected UAV aerial photographs (UAV SfM) was performed in the Pix4D mapper software (v 4.8.4) (Table 1). Based on the results of the analysis, digital terrain models (DTMs) were developed for each flight:

- 60NF digital terrain model (DTM_60NF_);
- 70NF digital terrain model (DTM_70NF_);
- 70F digital terrain model (DTM_70F_);
- 90F digital terrain model (DTM_90F_).

The *Pix4D mapper* software offers automatic point cloud classification. Due to the larger amount of biomass on the ground during this study (established forest order), it was noticed that, in some places, points were classified as ground, which was not true in reality. This software uses only points classified as ground or road for the development of a DTM. Since such places, in the case of insufficiently good classification, can cause possible errors, additional manual classification of point clouds was performed, based on 70F, and a new DTM (DTM_70MC_) was created. The time required for the manual classification of the point cloud was 7 h.

In further analysis, the GNSS survey was used as a reference measurement method.

A database was created in MS Excel, and the RMSE of spatial coordinates (x, y, xy, z, and xyz) of the terrain points was calculated according to the following Formula (2):

(2) $${RMSE}_{{coordinate}_{d}}=\sqrt{\frac{\sum {coordinate}_{d}^{2}}{n}}$$

where

${coordinate}_{d}$—spatial difference (x, y, xy, z, or xyz);

*n*—number of terrain points.

For an analysis of spatial coordinates’ RMSE, the same measurement points (their placement) for each survey method were used.

Next, the analysis of spatial coordinates’ error dependence on station (distance from the first profile) was conducted.

In addition to the RMSE of spatial coordinates, the recorded average cross-terrain slope for each profile was also analyzed (Figure 4). The average cross-terrain slope was calculated according to the following Formula (3):

(3) $$CTS=\frac{\delta H}{d_{h}}$$

where

*CTS*—cross-terrain slope (%);

*δH*—height difference (z);

$d_{h}$—horizontal distance between cross-section end points.

Finally, the deviation of the recorded points of the central axis was analyzed via the method used in the study by Kweon et al. [46]. The centerline points recorded by the theodolite, total station, and UAV SfM surveys were overlaid with the buffer zone around the GNSS centerline. The radius of the buffer zone was increased by 0.1 m until 100% of all survey points were inside it.

A statistical analysis was performed in IBM SPSS Statistics (v.29.0). Data normality testing was performed using a Kolmogorov–Smirnov test. One-way analysis of variance (ANOVA) followed by the Tukey post hoc test was used to determine the differences between terrain side slopes measured with the tested methods. A correlation and regression analysis was used to determine the relationship and connection between the cross-terrain slope and measurement error.

## 3. Results

The central axis of the planned forest road route recorded with the GNSS receiver was 879.42 m. The total station survey was the only method with a shorter recorded centerline (879.39 m). The difference in the central axis length recorded via the UAV SfM survey differed between +0.02 m (DTM_60NF_) and +0.13 m (DTM_70NF_). The height difference between the first and last points of the central axis measured with the GNSS receiver was 66.50 m. All UAV SfM surveys recorded smaller elevation differences between the first and last points. The classical survey method (level) and total station recorded higher elevation differences and greater longitudinal slopes versus the GNSS survey (Table 2).

The spatial accuracy of the recorded points (x, y, and z value) was tested on 250 points (Table 3). The largest spatial deviation (of all coordinates) was recorded for the classic survey method—using a theodolite and a level. All survey methods recorded higher RMSE values for the x-axis versus the y-axis. The UAV SfM DTM spatial errors were approximately the same (0.19–0.22 m). With manual classification of the point clouds, the z-coordinate RMSE value of DTM_70MC_ was reduced by 0.01 m compared with that of DTM_70F_.

**Figure 5:**
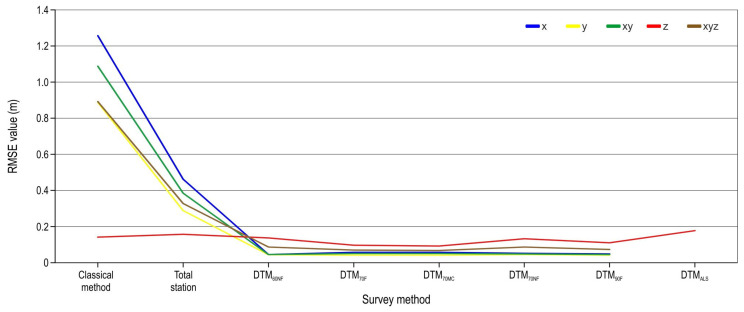
Coordinate RMSE by method (only centerline points).

The program used for classical survey method field data processing (*Cesta*) places cross-terrain points perpendicular to the central axis (points) of the road. Although special care was taken to record the cross-terrain points perpendicular to the central axis, certain deviations were found that affected the results of the analysis. For this reason, a spatial analysis of only the points that represented the central axis of the planned forest road (98 points) was carried out. The new analysis showed lower RMSE values for all survey methods (Figure 5). The classical survey method recorded the highest RMSE value for both the x (1.25 m) and y (0.89 m) coordinates. On the other hand, the smallest horizontal coordinate RMSE values were recorded for the UAV SfM survey: DTM_60NF_ (0.04 m) for the x coordinate and DTM_70F_ (0.04 m) for the y coordinate. The highest recorded z-coordinate RMSE values were calculated for DTM_ALS_ (0.18 m) and total station (0.16 m). The Z value RMSE of the UAV SfM survey ranged between 0.09 m (DTM_70MC_) and 0.14 m (DTM_60NF_). With manual classification of the point cloud (DTM_70MC_), the z-coordinate RMSE value was reduced by 0.01 m compared with that for the automatic classification of the same point cloud (DTM_70F_–0.10 m). The highest total spatial error (x, y, and z coordinate) was recorded for the classical survey method (0.89 m), and the lowest for DTM_70MC_ (0.07 m). The difference between the DTM_70F_ and the DTM_70NF_ total error RMSE was 0.02 m.

**Figure 6:**
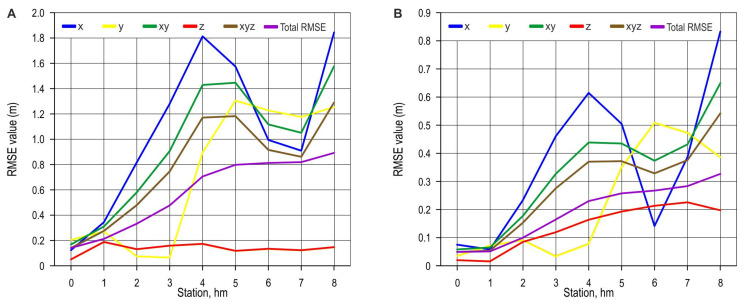
Coordinate RMSE by station: (**A**) classical survey; (**B**) total station survey.

**Figure 7:**
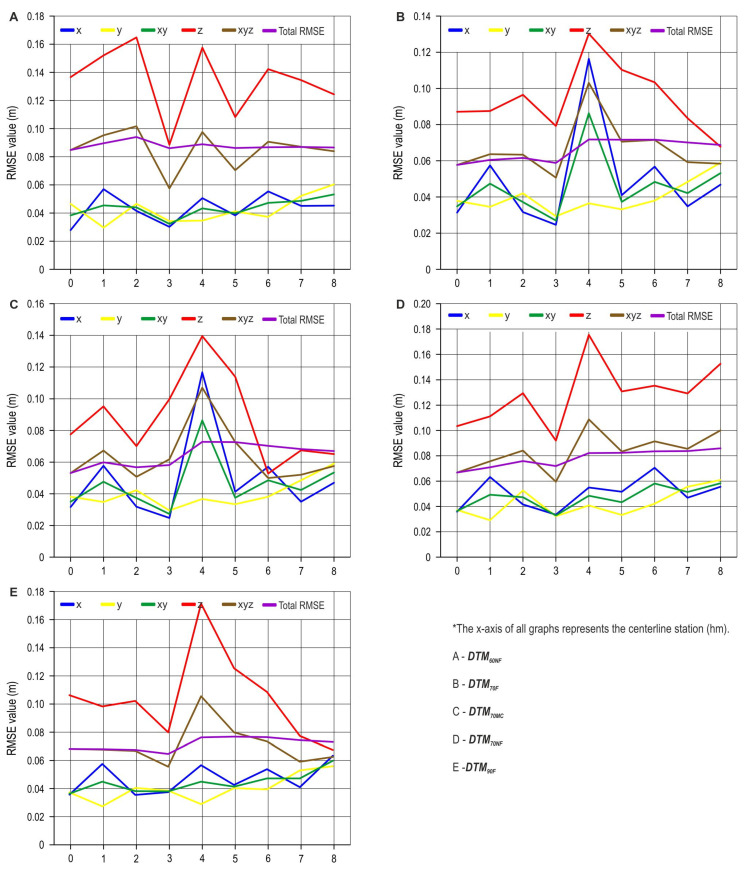
Coordinate RMSE by station—UAV SfM survey.

As some of the survey methods require frequent relocation of the measuring device (Figure 3), an analysis of the spatial errors by station (Figure 6 and Figure 7) was performed. The total RMSE value of the coordinates was constantly increasing for the classical survey method and the total station (Figure 6). The highest x-coordinate RMSE values were recorded for field points at the station between 3+00.00 hm and 5+00.00 hm and, on the final part of the planned road, after 7+00.00 hm, for both the classic survey method and the total station. The deviation of the mentioned methods for the *y*-axis takes on values greater than 0.1 m after 3+00.00 hm for the classical survey method and from 4+00.00 hm for total station survey. The lowest z-coordinate RMSEs of the classical survey method were recorded between 0+00.00 and 1+00.00 hm (0.05 m), and the highest between 1+00.00 hm and 2+00.00 hm (0.19 m). The values did not exhibit an upward trend after the 2+00.00 hm station. The total station z value errors had an upward trend between 2+00.00 hm and 7+00.00 hm, where the maximum z-coordinate RMSE (0.23 m) was recorded.

Looking at the horizontal accuracy (x and y coordinates) of the UAV SfM survey, evidently, the RMSE ranged between 0.03 and 0.07 cm along the whole route (all stations) for all DTMs except DTM_70F_ and DTM_70MC_, where a larger deviation was observed on the part of the route with the 4+00.00 hm station (Figure 7). A larger vertical deviation (z coordinates) was observed between the 4+00.00 hm and 5+00.00 hm station. DTM_60NF_ achieved the largest deviations in most sections of the planned route. The highest RMSE of an individual section of the route was recorded with DTM_70NF_ at 4+00.00 hm (0.18 m). Although a manual classification of the point cloud was carried out for the DTM_70MC_ creation, at the station between 4+00.00 hm and 7+00.00 hm, larger z value deviations were recorded compared with that for DTM_70F_.

The average cross-terrain slope was calculated for each profile on the planned route of the forest road (Table 4). The cross-terrain slope (measured with the GNSS receiver) ranged from a minimum of 1.14% to a maximum of 58.96%, while the average cross slope along the entire route was 31.58%. The largest deviation of the average cross-terrain slope was obtained for the UAV SfM surveys (between 32.11 and 32.15%). The highest RMSE was obtained for the classical survey method (3.12%), while the lowest was obtained for the total station survey (0.27%).

**Figure 8:**
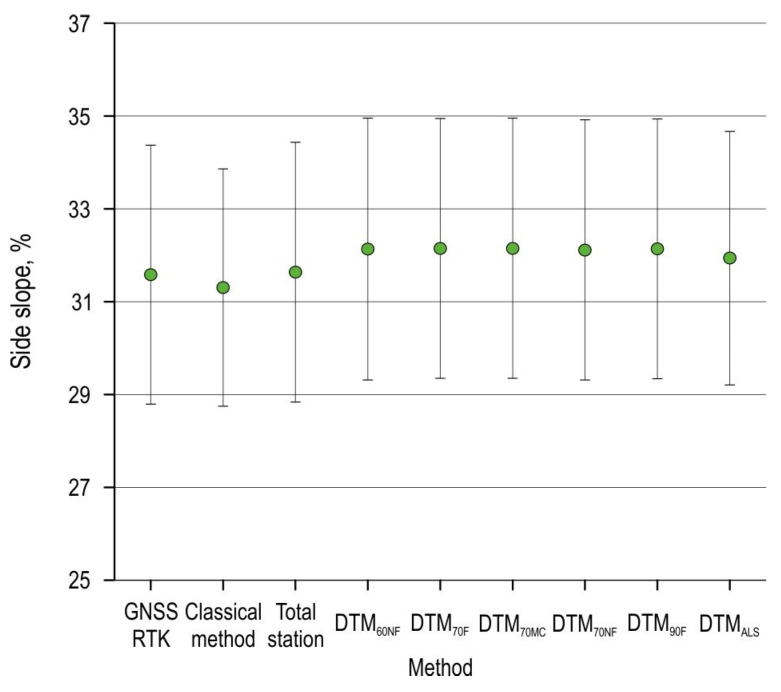
Difference in measured terrain side slope by method. The data is presented as the mean ± SE (standard error).

The cross-terrain slope data was normally distributed, as the Kolmogorov–Smirnov and Shapiro–Wilk tests showed significance level >0.05. An ANOVA followed by post hoc Tukey HSD (honestly significant difference) test indicated no statistically significant differences for the measured side slopes between survey methods (Figure 8).

**Figure 9:**
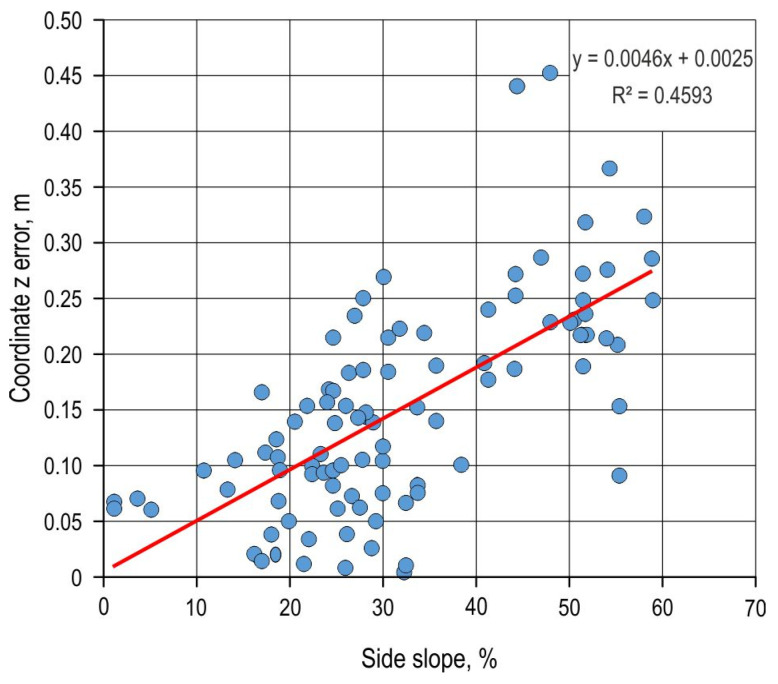
Impact of side slope on the z-coordinate error of DTM_ALS._

For all survey methods, a correlation and regression analysis of the influence of the cross-terrain slope on the measurement error was performed. For the z-coordinate error of the DTM_ALS_ and cross-terrain slope, the Pearson correlation coefficient (r) was 0.678, with the equation y = 0.0046x + 0.0025, indicating a moderate correlation between the observed variables according to the Chaddock scale [56] (Figure 9). The coefficient of determination (R^2^) was 0.4593. The Pearson correlation coefficient between the z-coordinate error of the total station survey z coordinates and cross-terrain slope was 0.336, with R^2^ = 0.103. The correlation analysis showed a negligible correlation (r = 0–0.3), i.e., R^2^ < 0.04, between other survey methods, regardless of the tested coordinate.

**Figure 10:**
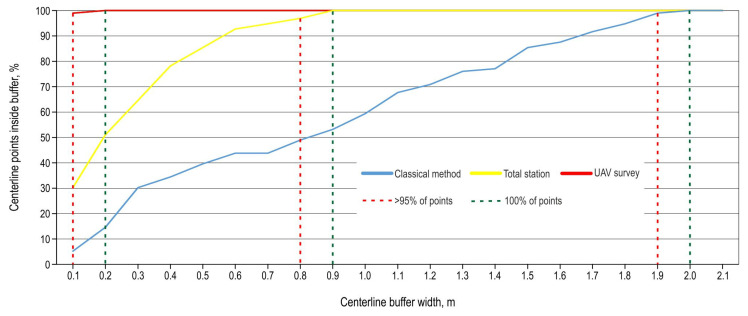
Offset of off-road centerline points.

A deviation analysis of the central axis points recorded with different survey methods from the central axis recorded using the GNSS receiver showed that, for all UAV SfM surveys, more than 95% of the points were within 10 cm and 100% of the points were within 20 cm of the GNSS centerline (Figure 10). The classical survey method points required the largest buffer width (1.9 m for 95% of the points or 2.0 m for 100% of the points).

## 4. Discussion

This study tested the spatial accuracy of the survey methods used for field data collection, which is necessary for forest road design. At longer distances (in this study, the total length of the route was 879.42 m), a greater deviation of the classical survey method (theodolite and level) and the total station from the GNSS receiver was found. The constant increase in the spatial coordinate RMSE values of the classical survey method and the total station when moving away from the first point indicates a collimation error (centering and levelling) of the instrument. Although much attention was paid to the collimation and placement of the device (the theodolite and total station), due to field conditions (loose soil with rock elements), the instrument inevitably moved and was displaced. Although these displacements were not large, due to the cumulative addition of their errors, the total spatial RMSE was 0.89 m for the classical survey method and 0.33 m for the total station. In forest conditions, in order to ensure the visibility of all points, frequent changes in instrument position are inevitable. These changes in instrument position can significantly affect the quality of the field survey. As the research goal was to test the accuracy of survey equipment in forest conditions, no GCPs were chosen for total station correction. The total station should be the most accurate survey equipment when corrections are made (GCP), but in some forest areas, for example, selection forests, where species such as Norway spruce (*Picea abies* (L.) Karst.) and European silver fir (*Abies alba* Mill.) dominate, obtaining reliable GNSS surveys (for GCP placement) and using the full potential of a total station is very difficult. For this reason, no GCPs were used for the total station survey.

The UAV SfM xyz-coordinate RMSE ranged between 0.07 and 0.09 m. The RMSE values obtained from measurements using UAVs are in accordance with those obtained by other authors [33,57,58]. Of all the coordinates, the largest deviation was determined for the z coordinate (from 0.09 m (DTM_70MC_) to 0.14 m (DTM_60NF_)). Part of this error can certainly be explained by the biomass left on the ground at the time of the survey. This claim can be confirmed by comparing the RMSE values from the analysis of all points and those of the points on the central axis (where no biomass was found). The authors conclude that different flight altitudes did not affect the spatial accuracy as much as the terrain follow flight option did, especially for the z coordinate. Between DTM_70F_ and DTM_90F_, the difference between the achieved RMSE values was minimal (0.01 m for the z coordinate), while between DTM_70F_ and DTM_70NF_, it was greater (0.03 m). For horizontal coordinates (x and y), the RMSE difference did not exceed 0.01 m, regardless of flight altitude or terrain follow option. The z-coordinate RMSE was reduced by 0.01 m by manually classifying the point clouds. The largest difference between DTM_70F_ and DTM_70MC_ for a single point of analysis was 0.27 m, but the total average difference was 0.003 m. At some check points, a higher altitude was also determined for DTM_70MC_ (the greatest difference was 0.07 m). The higher altitudes of some check points can be explained by the lack of data (after removing points that are not ground) necessary for quality interpolation of the terrain. The authors conclude that manual classification gives better spatial results only when smaller areas are excluded, especially if the terrain relief is more pronounced and complex. Based on the obtained results, the following question arises: Is it necessary to carry out manual classification? Considering the spatial accuracy of the points exclusively, the 7 h required for manual classification certainly did not give the expected results. To draw a conclusion about the need for manual classification, this procedure needs to be examined when calculating the amount of earthwork required for the construction of a lower structure. In addition to manual classification, the use of various forms of machine learning and a combination of lidar and photogrammetric data should also be addressed in future research [59].

Regardless of the flight altitude and the terrain follow option, larger deviations from the UAV SfM coordinates were recorded on the part of the route between the 4+00.00 hm and 6+00.00 hm station. In this section, the amount of biomass on the ground was the same as in other sections. A possible reason for these deviations is the proximity of the powerline above, which forced the UAV to change directions. This hypothesis was confirmed by the horizontal deviation of the coordinates read from DTM_70F_, based on flight 70F, which was closest in altitude to the powerline in the mentioned section of the planned forest road route. When planning the flight mission, the recommendations for the UAV’s flight near the powerline were followed [60]. The authors conclude that further research on this issue is necessary.

The DTM_ALS_ z-coordinate RMSE was 0.24 m for the analysis with all points and 0.18 m for the analysis with points on the central line. The greater z-coordinate deviations compared with those from the UAV SfM survey can be explained by the smaller number of points recorded per m^2^ [61]. The tested lidar data had an average of 4 points per m^2^ [55], while the number of points per m2 for UAV SfM was >120. The main advantage of the lidar system over SfM is the possibility of recording the terrain (ground) under a dense canopy set, i.e., the possibility of recording throughout the whole year in deciduous stands, which is not possible for photogrammetry-based surveys [62,63]. The authors conclude that lidar data of the tested characteristics can be used for the planning of forest roads, while their use in design will be tested in future research. A high R^2^ value (0.4593) indicates a strong relationship between the side slope and z-coordinate error. This result is consistent with that obtained by other authors [64,65]. Unfortunately, the time of lidar scanning and the equipment used are not available to the public. If they were, the accuracy analysis could be of better quality.

Although the values of the z-coordinate RMSE ranged between 0.19 m and 0.47 m for the tested survey methods, no statistically significant difference was found between the recorded cross-terrain slopes. As the value of the cross-terrain slope is one of the main factors influencing the amount of earthworks and consequently the cost of construction [47,48], the large coordinate RMSE values achieved do not necessarily mean that the data obtained through other methods are not usable when designing forest roads. For this reason, an analysis of the impact of z-coordinate RMSE on the differences in the amount of earthwork that will occur on the designed forest road needs to be conducted to determine or refute the existence of a mutual relationship.

By analyzing the deviation of the central axis points recorded via the total station from the central axis recorded using the GNSS receiver, the buffers needed to contain 95% and 100% of points were consistent with the research of Kweon et al. [46] (0.94 and 0.95 m). In the mentioned research, the authors analyzed the accuracy of the total station not only at a larger total length of forest road central axis but also in a longer interval between the recorded points (20 m interval).

All tested methods achieved an accuracy that allows for quality forestry road planning. As salvage logging was conducted on the researched area, terrain surveys with GNSS devices and UAV SfM were possible. In different forest conditions (dense canopies of selected forests and regular forests under leaf-on condition), GNSS and UAV SfM surveys would be questionable or would have reduced accuracy [66,67]. On the other hand, UAV SfM DTM in leaf-off forest conditions can achieve relatively high accuracies [68] and be used for planning. A total station combined with GCPs in certain conditions could be more practical than UAV SfM (forests composed of deciduous tree species throughout a leaf-off season).

Even though planning is possible with the achieved accuracy, the accuracy needed for forest road design needs to be tested based on measurable parameters such as calculated earthwork, width, and slope in different forest and relief conditions.

## 5. Conclusions

Based on the conducted research, the authors draw the following conclusions:

- At field points with higher stations, values recorded using the classical survey method (with a theodolite and a level) and a total station show an accumulation of errors due to an increased number of changes in the instrument position. By reducing the number of instrument stations, errors can be reduced. Unfortunately, in forest conditions and when surveying complex terrains, this condition cannot always be met.
- The UAV SfM provides data with high spatial accuracies. Spatial accuracy is influenced by vegetation, i.e., biomass on the ground. Terrain follow flight image-based DTMs achieve greater spatial accuracy than those without the flight option.
- Although manual classification of point clouds can produce more accurate data, especially using the z coordinate, in some cases, it can also cause less accurate DTMs than the one created based on automatic classifications. Such phenomena have been found in places where most of the point cloud has been erased.
- The ALS survey achieved a higher z-coordinate RMSE compared with that of the UAV SfM survey, even though it has a higher penetration of the tree canopy assembly and vegetation. The ALS z error was strongly influenced by the cross-terrain slope. The authors conclude that the tested lidar data can be used for forest road planning, while their use in design should be examined in further research.
- Although no differences were found between cross-terrain slopes recorded using different survey methods, further analyses and research on the applicability of field data collected via these methods need to be conducted and their impact on other forest road design processes needs to be tested.

## Figures and Tables

**Table 1:** Flight parameters and results of the photogrammetric analysis.

	60NF	70NF	70F	90F
Flight altitude	60 m	70 m	70 m	90 m
Terrain follow	No	No	Yes	Yes
Number of aerial photographs	827	666	692	414
Front/side overlap	80/80%	80/80%	80/80%	80/80%
Area	24.20 ha	26.54 ha	27.89 ha	29.64 ha
GSD *	2.32 [cm/pixel]	2.66 [cm/pixel]	2.28 [cm/pixel]	2.86 [cm/pixel]
DTM resolution	5 × GSD (2.32 [cm/pixel])	5 × GSD (2.66 [cm/pixel])	5 × GSD (2.28 [cm/pixel])	5 × GSD (2.86 [cm/pixel])
Processing time (total)	6 h 22 min	4 h 42 min	5 h 54 min	4 h 22 min

* GSD—distance between two consecutive pixel centers measured on the ground.

**Table 2:** Recorded centerline length, elevation difference, and longitudinal terrain slope by method.

Survey Method	Centerline Length (m)	Elevation Difference (m) Between First and Last IPs	Average Longitudinal Terrain Slope (%)
**GNSS**	879.42	66.50	7.56
**Classical**	880.51	66.70	7.57
**Total station**	879.39	66.74	7.59
**DTM_60NF_**	879.44	66.42	7.55
**DTM_70F_**	879.48	66.46	7.56
**DTM_70MC_**	879.48	66.45	7.56
**DTM_70NF_**	879.55	66.37	7.55
**DTM_90F_**	879.53	66.49	7.56

**Table 3:** Coordinate RMSE values by survey method.

Coordinate	Classical Method (m)	Total Station (m)	DTM_60NF_ (m)	DTM_70F_ (m)	DTM_70MC_ (m)	DTM_70NF_ (m)	DTM_90F_ (m)	DTM_ALS_ (m) *
x	1.72	0.45	0.10	0.10	0.10	0.11	0.12	/
y	1.58	0.28	0.09	0.09	0.09	0.09	0.09	/
z	0.47	0.15	0.22	0.20	0.19	0.21	0.20	0.24

* As the ALS survey was conducted before the experiment was set (absence of ground check point markings), the horizontal accuracy could not be determined.

**Table 4:** Descriptive statistics of the cross-section side slope by method.

	GNSS	Classical	Total Station	DTM_60NF_	DTM_70F_	DTM_70MC_	DTM_70NF_	DTM_90F_	DTM_ALS_
**Average (%)**	31.58	31.31	31.64	32.13	32.15	32.15	32.11	32.13	31.94
**Max (%)**	58.96	57.69	59.23	58.80	58.76	58.75	59.25	59.20	63.01
**Min (%)**	1.14	2.19	0.89	1.11	1.25	1.30	1.39	1.58	3.23
**Median (%)**	28.12	27.56	28.08	28.83	28.84	29.00	28.90	28.85	28.66
**RMSE (%)**	/	3.12	0.27	1.87	1.80	1.77	1.81	1.81	2.11

## Data Availability

The data is contained within this article.
